# Zebrafish biosensor for toxicant induced muscle hyperactivity

**DOI:** 10.1038/srep23768

**Published:** 2016-03-31

**Authors:** Maryam Shahid, Masanari Takamiya, Johannes Stegmaier, Volker Middel, Marion Gradl, Nils Klüver, Ralf Mikut, Thomas Dickmeis, Stefan Scholz, Sepand Rastegar, Lixin Yang, Uwe Strähle

**Affiliations:** 1 Institute of Toxicology and Genetics, Karlsruhe Institute of Technology (KIT), Postfach 3640, D76021 Karlsruhe; 2 Department of Environmental Pollution and Health, State Key Laboratory of Environmental Criteria and Risk Assessment, Chinese Research Academy of Environmental Sciences, 100012, Beijing, China; 3 Institute for Applied Computer Sciences, Karlsruhe Institute of Technology (KIT), Postfach 3640, D76021 Karlsruhe, Germany; 4 Department of Bioanalytical Ecotoxicology, UFZ – Helmholtz Centre for Environmental Research, D04318 Leipzig, Germany; 5 Faculty of Biosciences, Ruprecht-Karls-University of Heidelberg, D69120 Heidelberg, Germany

## Abstract

Robust and sensitive detection systems are a crucial asset for risk management of chemicals, which are produced in increasing number and diversity. To establish an *in vivo* biosensor system with quantitative readout for potential toxicant effects on motor function, we generated a transgenic zebrafish line *TgBAC*(*hspb11:GFP*) which expresses a GFP reporter under the control of regulatory elements of the small heat shock protein *hspb11*. Spatiotemporal *hspb11* transgene expression in the musculature and the notochord matched closely that of endogenous *hspb11* expression. Exposure to substances that interfere with motor function induced a dose-dependent increase of GFP intensity beginning at sub-micromolar concentrations, while washout of the chemicals reduced the level of *hspb11* transgene expression. Simultaneously, these toxicants induced muscle hyperactivity with increased calcium spike height and frequency. The *hspb11* transgene up-regulation induced by either chemicals or heat shock was eliminated after co-application of the anaesthetic MS-222. *TgBAC*(*hspb11:GFP*) zebrafish embryos provide a quantitative measure of muscle hyperactivity and represent a robust whole organism system for detecting chemicals that affect motor function.

The increasing diversity of chemicals produced by industry generates concerns regarding the potential hazards on biota and human health. The European Union’s REACH (Registration, Evaluation, Authorization and Restriction of Chemicals) regulation declares that the commercial producers are responsible for risk assessment and management of the chemicals that they produce. A major emphasis is placed on chemical safety requiring the evaluation of potential adverse toxicological outcomes and ecotoxicological hazards prior to selling the chemicals. Embryos of zebrafish (*Danio rerio*) were shown to be efficient for such toxicological estimation showing, for instance, a correlation and equal sensitivity to acute fish toxicity^1^,^2^.

Molecular endpoints derived from toxicogenomic responses provided other endpoints that allow us to demonstrate the presence of compounds with a specific, potentially tissue-specific mode of action^3^,^4^,^5^,^6^. This observation has led to the development of transgenic biosensor lines that utilize genetic regulatory elements to drive the expression of fluorescent proteins and thereby report the presence of target classes of substances in the environment, such as estrogenic compounds^7^,^8^,^9^,^10^, dioxins^11^, heavy metals^12^,^13^,^14^, goitrogens^15^ and glucocorticoids^16^,^17^,^18^. Interestingly, many of these biosensor genes exhibit tissues preferably responsive to particular classes of pollutants^6^. Although there are approaches using tissue-specific fluorescent reporter lines for the testing of chemical compound effects on, for instance, the heart, blood vessels, central nervous system and pancreas^19^, interpretation of results may not be straightforward. Many substances affect motility and thereby indirectly survival. This prompted us to develop a biosensor system that provides simple and quantifiable readouts for effects of chemicals on the skeletal musculature that combines physiological with behavioural readouts.

Heat shock proteins (HSPs) are known for their immediate responses upon exposure to a wide range of chemical stressors with little dependency on chemical structures^3^,^13^. Interestingly, we found that one of the vertebrate HSP family genes, *hspb11*, becomes up-regulated specifically by chemicals that interfere with motor function^20^. The up-regulation was discussed as a potentially protective response upon over-excitation of the muscle and increase of intracellular Ca^2+^ levels in the muscle cells. We therefore investigated whether *hspb11* gene regulatory elements can be used to build a biosensor to detect chemicals that impair motor function.

Motor dysfunction can be caused by problems in motor neurons and/or in the muscle. At the neuromuscular junction, a neurotransmitter, acetylcholine (ACh), is released from pre-synaptic terminals of motor neurons. Upon ACh binding to the post-synaptic nicotinic ACh receptor complex, the membrane of the muscle end plate depolarizes rapidly. This depolarization activates a cluster of voltage-gated sodium channels at the neuromuscular junctions and converts local end plate depolarization into a muscle fibre-wide action potential^21^. The action potential triggers a cascade of events that leads to muscle contraction, mediated by transient release of calcium ions from the sarcoplasmic reticulum. Because chemical interference with each of these distinct processes affects motility, we require assays which report the mode of action.

We report here that the bacterial artificial chromosome (BAC) transgenic zebrafish *TgBAC*(*hspb11:GFP*) line specifically responded to chemicals that interfere with motor function and that cause induction of muscle hyperactivity. Dose-dependent up-regulation of *hspb11* transgene expression was observed with a structurally wide range of chemicals and correlated with motility defects. We demonstrate that *hspb11* transgene expression is induced specifically by muscle hyperactivity irrespective of the state of muscle integrity.

## Results

### A zebrafish *hspb11* BAC transgenic line recapitulates the expression of the endogenous gene

We previously reported that *hspb11* transcription was significantly induced by the pesticide, azinphosmethyl which impairs muscle integrity^20^. As no biosensor system was available for *in vivo* detection of chemicals causing motor dysfunction, we aimed at developing a transgenic zebrafish reporter line recapitulating the basal and induced expression patterns of *hspb11*. For this purpose, we obtained a BAC clone that includes the *hspb11* locus and inserted a GFP reporter into the single exon locus of *hspb11* by homologous recombination in *E. coli*^22^,^23^. To facilitate the insertion of the recombined BAC fragment with GFP reporter into the zebrafish genome, we also introduced the iTol2 cassette into the BAC backbone for Tol2 transposase mediated insertion^24^.

**Figure 1:**
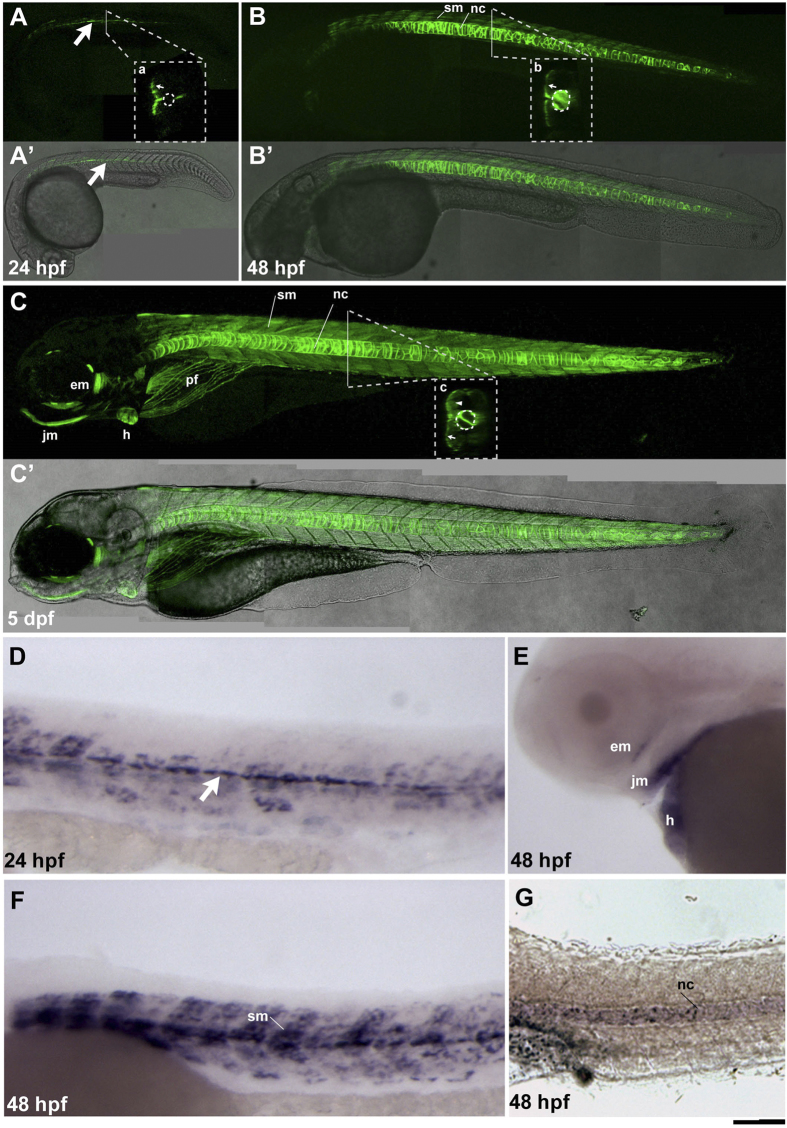
Zebrafish *hspb11* BAC transgenic line recapitulates the endogenous gene expression. Expression of the *hspb11* transgene (GFP, green) at 24 hpf (**A–A’**), 48 hpf (**B–B’**) and 5 dpf (**C–C’**). Lateral views are shown with the anterior end oriented to the left. Insets (a-c) show optical transverse sections, dorsal side up. Fluorescence channel image of the *hspb11* reporter (**A–C**) is merged with the respective bright field transmission images (**A’–C’**). (**A–A’**) *hspb11* transgenic embryos at 24 hpf show GFP reporter expression in the muscle cells (big arrow) in a layer of slow muscles (inset a, small arrow). A stippled circle in the inset (a) represents the notochord. (**B–B’**) At 48 hpf, stronger GFP expression appears in the notochord (nc, also inset b dotted circle) and in the slow muscle cells (sm; a small arrow in the inset b). (**C–C’**) At 5 dpf the GFP expression in slow muscle cells (sm) and notochord (nc) persists, with additional expressions in the heart (h), eye muscles (em), pectoral fins (pf), jaw muscles (jm) and weakly in the fast muscles (c, arrowhead). (**D–G**) *In situ* gene expression analysis of *hspb11* at 24 (D) and 48 hpf (**E–G**) in wildtype embryos. At 24 hpf, *hspb11* expression appears in the muscle pioneer cells (**D**, white arrow), and it is further expressed in the slow muscles at 48 hpf (**F**, sm). (E) At 48 hpf *hspb11* expression is also seen in the muscle of the eye (em), the jaw muscles (jm) and in the heart (h). (G) *hspb11* expression is visible in the notochord at 48 hpf. Scale bar: (**A–C’**) 200 μm, (**D–G**) 100 μm.

The established *TgBAC*(*hspb11:GFP*) transgenic line recapitulated the endogenous *hspb11* expression pattern throughout embryonic development. At 24 hours post-fertilization (hpf), GFP-positive cells were detected in muscle pioneer cells at 24 hpf (Fig. 1A–A’, big arrow). At 48 hpf, the *hspb11* transgene is expressed in the slow muscles (Fig. 1B–B’, sm). Robust expression of the GFP reporter was also seen in the notochord at 48 hpf and later stages (Fig. 1B–B’, b and C–C’, c). At 5 days post-fertilization (dpf), GFP was detected in slow muscle fibres, the notochord, the heart, jaw and eye muscles and pectoral fins (Fig. 1C–C’). This expression pattern of the *hspb11* transgene is consistent with the endogenous *hspb11* gene expression pattern (Fig. 1D–G). Moreover, heat shock activated expression of the transgene mRNA in slow and fast muscles to a similar levels as the endogenous *hspb11* gene (data not shown). Thus, we established a *TgBAC*(*hspb11:GFP*) line that faithfully reflects the endogenous gene expression with correct spatial and temporal distribution.

### The *hspb11* transgene responses to pesticides

**Figure 2:**
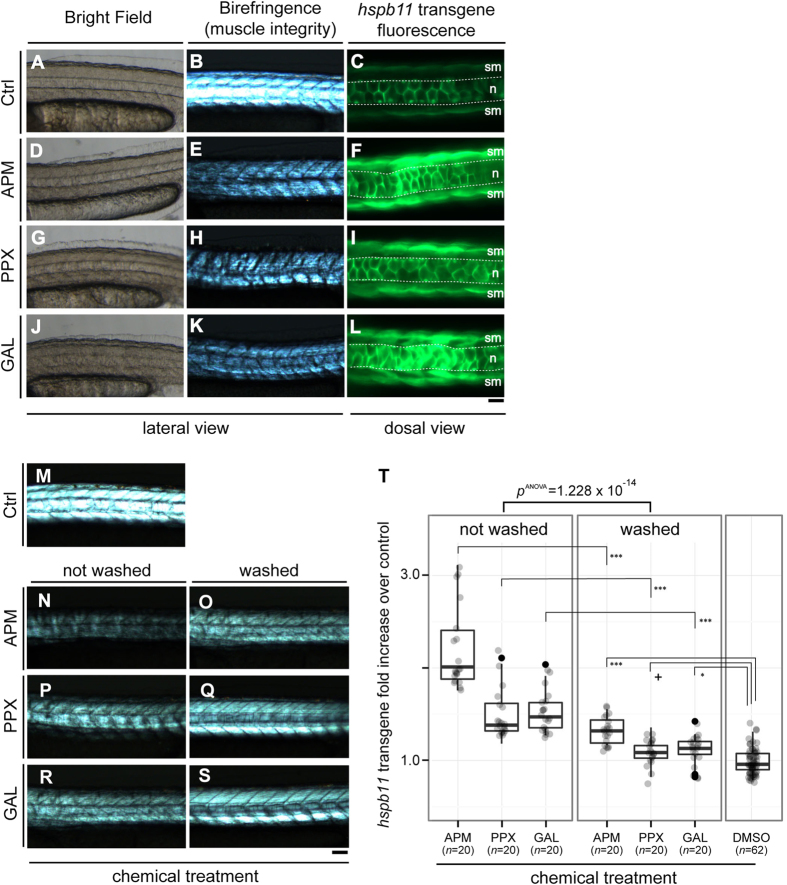
The *hspb11* transgene responses to pesticides. Embryos from *TgBAC*(*hspb11:GFP*) were treated with either DMSO (solvent control; **A–C** and **M**), or the pesticides azinphosmethyl (APM; **D–F**), propoxur (PPX; **G–I**) or galanthamine (GAL; **J–L**). The embryos were treated from 90%-epiboly (9 hpf) to 48 hpf and examined for birefringence as a readout of muscle integrity (**B,E,H,K**; lateral view of the trunk region) and *hspb11* transgene expression (**C,F,I,L**; dorsal view of the trunk region). Corresponding bright field images are given in (**A,D,G,J**; lateral view). The stippled lines (in **C,F,I,L**) delineate the notochord (n). sm: slow muscle. Anterior left. (**M–S**) Embryos from *TgBAC*(*hspb11:GFP*) were treated with either azinphosmethyl (**N,O**), propoxur (**P,Q**) or galanthamine (**R,S**). The *hspb11* transgene level was compared between two groups: one group treated with chemicals from 9–48 hpf (“not washed”; **M,N,P,R**), another treated from 9–24 hpf, followed by several washes in fresh fish water until 48 hpf (“washed”; **O,Q,S**). The intensity of *hspb11* transgene is expressed in fold increase over control. Scale bars: (**A,B,D,E,G,H,J,K,M–S**) 100 μm; (**C,F,I,L**) 50 μm. (**T**) Quantification of the experiments exemplified in (**M–S**). The intensity of *hspb11* transgene expression is shown as fold induction over DMSO control. There were significant effects of the washout of the compounds on the *hspb11* transgene level (one-way ANOVA, *F*[1, 118]* *= 77.762, *p *= 1.228 × 10^−14^), with azinphosmethyl (TukeyHSD test from here onward, *p *= 0.0000000), propoxur (*p *= 0.0000429), and galanthamine (*p *= 0.0000087) treated embryos now showing lower *hspb11* transgene expression. However, all washed groups showed still higher *hspb11* transgene levels in comparison to DMSO solvent control (one-way ANOVA, *F*[3, 118]* *= 29.316, *p *= 3.09 × 10^−14^; TukeyHSD test, ***p = 0.0000000 for azinphosmethyl vs. control, *p *= 0.0507833^+^ with a substantial difference for propoxur vs. control, and *p = 0.0124261 for galanthamine vs. control).

To test whether the transgene expression is responsive to compounds that cause muscle toxicity, we treated *TgBAC*(*hspb11:GFP*) embryos with azinphosmethyl, propoxur and galanthamine, which exert their toxic effects on muscle through inhibition of Acetylcholine esterase (AChE) activity^20^. Embryos were exposed to the three pesticides from the 90%-epiboly stage (9 hpf) to 48 hpf. Treatments with all three compounds caused an up-regulation of GFP expression in the slow muscle (sm, Fig. 2F,I,L) and also in the notochord (n, Fig. 2F,I,L) compared with a negative control treated with 0.1% (v/v) DMSO only (Fig. 2C). To assess muscle fibre arrangement or integrity under these treatments, we examined birefringence of the muscles. We observed that all chemicals also caused a reduction in muscle birefringence, consistent with their previously described effects and paralleling the transgene response. The magnitude of the transgene expression was roughly proportional to the loss of muscle integrity observed via the assessment of birefringence. We also assessed whether transgene activation is restricted to slow muscle by *in situ* hybridisation of azinphosmethyl treated and DMSO control embryos. An increase of *gfp* mRNA was detected in both slow and fast muscles in azinphosmethyl treated embryos but not solvent controls (data not shown).

To further ask whether the transgene expression level correlates with the reduced muscle integrity, we examined whether the transgene expression level would be reversed after recovery of muscle integrity by washing the compounds off. We compared GFP reporter levels between two groups for each of the three chemicals; one group treated with the chemical from 9–48 hpf, while another group treated from 9–24 hpf, followed by several washes in fresh fish water until 48 hpf (Fig. 2M–T). For all chemical treatments, washout of the chemicals led to higher birefringence (Fig. 2N–S). This recovery of muscle integrity after the washout of the compounds was accompanied by a reduction of *hspb11* transgene expression levels (Fig. 2T, one-way ANOVA, *p *= 1.228 × 10^−14^). Consistent with a partial and not full recovery of muscle integrity assessed by birefringence, *hspb11* transgene levels after the washout were higher than those of DMSO negative control for two of the three chemical treatments (Fig. 2T; one-way ANOVA, *p *= 3.09 × 10^−14^; TukeyHSD test, *p *= 0.0000000 for azinphosmethyl vs. control, *p *= 0.0507833 for propoxur vs. control, and *p *= 0.0124261 for galanthamine vs. control).

### The *hspb11* transgene was specifically induced by chemicals that impair muscle integrity in a dose-dependent manner

We tested whether the *hspb11* transgene responds in a dose-dependent manner. We quantified the GFP reporter intensity in the trunk region (slow muscle + notochord) of *TgBAC*(*hspb11:GFP*) embryos after treatments with 14 test chemicals that have previously been shown to impair motor function. These chemicals exert their motor-toxic effects through inhibition of AChE, modulation of channel properties or other pathways (Table S1 and Fig. S3). All these chemicals induced impaired motility in a touch response assay (Table S2, Fig. S1). This was not simply due to general lethality, since impaired motility occurred already at doses well below the LC50 (Table S2, Fig. S2). We exposed the *TgBAC*(*hspb11:GFP*) embryos to these chemicals from 9–48 hpf and performed a semi-automatic quantitative assessment of GFP reporter intensity^25^.

**Figure 3:**
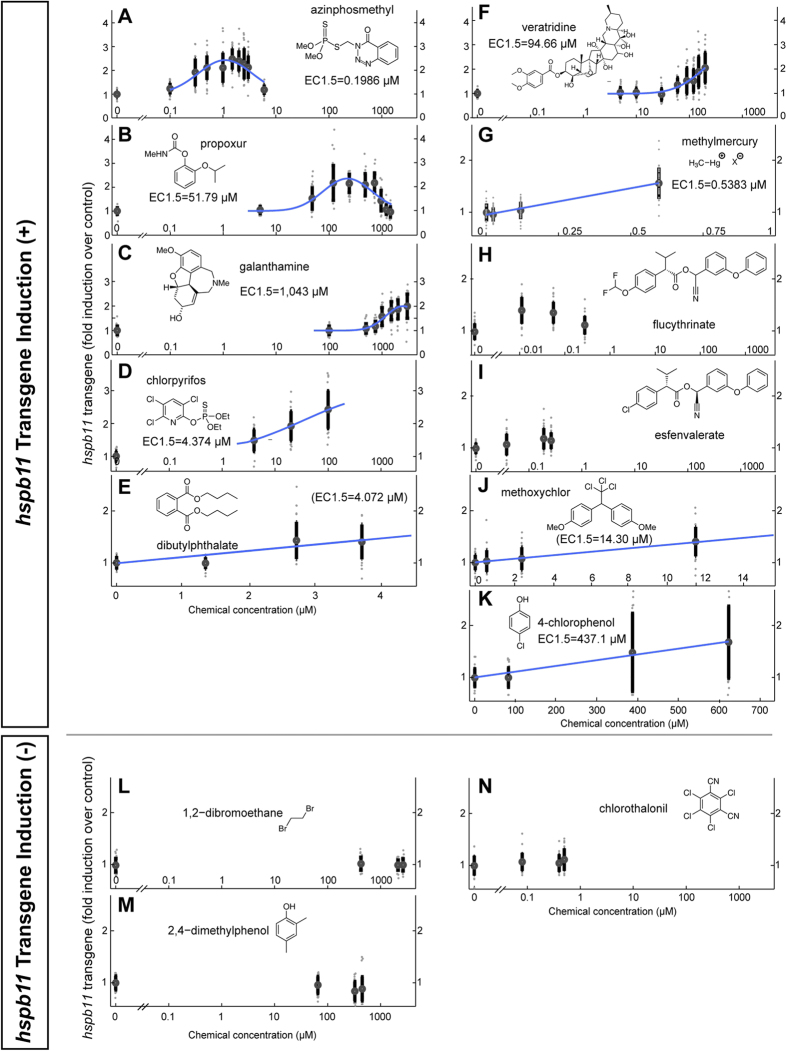
Dose-dependent and chemical-specific up-regulation of *hspb11* transgene expression. Embryos from *TgBAC*(*hspb11:GFP*) were treated with the following chemicals: azinphosmethyl (**A**), propoxur (**B**), galanthamine (**C**), chlorpyrifos (**D**) and dibutylphthalate (**E**), veratridine (**F**), methylmercury (**G**), flucythrinate (**H**), esfenvalerate (**I**), methoxychlor (**J**) and chlorophenol (**K**), dibromoethane (**L**), dimethylphenol (**M**) and chlorothalonil (**N**). The *hspb11* reporter levels were expressed in fold induction over control as a function of nominal chemical concentration (μM). Concentration dependent responses to the chemicals with up-regulation of the *hspb11* reporter level (one-way ANOVA *p *< 0.001) were modelled using a logarithmic Gaussian model, except methylmercury, dibutylphthalate, methoxychlor and 4-chlorophenol for which a linear model was used. The *hspb11* transgene level 1.5-fold increased over control is indicated by stippled horizontal lines in red. The EC1.5 dose (vertical stippled red lines) and modelled concentration-response curves (blue) were overlaid on the individual data points with mean (black circle) and standard deviation (vertical black bar) shown at each dose. The EC1.5 values of methoxychlor and dibutylphthalate in parenthesis are estimated values from linear modelling. Albeit their significant *hspb11* transgene responses, flucythrinate and esfenvalerate were not efficiently modelled (one-way ANOVA, *F*(3, 116)* *= 6.1961, *p *= 6.0 × 10^−4^, *F*(3, 117)* *= 26.0963, *p *= 5.369 × 10^−13^, respectively). One-way ANOVA values for the rest are the following: azinphosmethyl, *F*(9, 310)* *= 44.3539, *p *= 1.121 × 10^−50^; propoxur, *F*(9, 278)* *= 51.1080, *p *= 6.485 × 10^−54^; galanthamine, *F*(7, 245)* *= 56.6725, *p *= 9.363 ×  × 10^−48^; chlorpyrifos, *F*(3, 121)* *= 68.4166, *p *= 6.111 × 10^−26^; di-*n*-butylphthalate, *F*(3, 121)* *= 25.4847, *p *= 7.555 × 10^−13^; veratridine, *F*(8, 280)* *= 29.0567, *p *= 8.135 × 10^−33^; methylmercury, *F*(3, 118)* *= 58.4852, *p *= 3.071 × 10^−23^; methoxychlor, *F*(3, 119)* *= 21.7458, *p *= 2.681 × 10^−11^; 4-chlorophenol, *F*(3, 107)* *= 12.2473, *p *= 5.969 × 10^−7^; 1,2-dibromoethane, *F*(3, 109)* *= 0.3508, *p *= 0.788; 2,4-dimethylphenol, *F*(3, 121)* *= 4.5156, *p *= 4.0 × 10^−3^; chlorothalonil, *F*(3, 117)* *= 2.0164, *p *= 0.115.

AChE inhibitors impair muscle function by preventing the degradation of ACh at the neuromuscular junction. Genetic mutation of AChE function in the zebrafish severely disrupts muscle integrity as a result of muscle hyperactivity, and it also increases *hspb11* expression^20^,^26^. In line with these findings, a dose-responsive increase of *hspb11* transgene expression was detected after exposure of embryos to the AChE inhibitors azinphosmethyl, propoxur^27^, galanthamine, and chlorpyrifos (Fig. 3A–D). Consistent with the reported weak AChE inhibiting effect of dibutylphthalate^28^, we observed only weak effects on *hspb11* transgene expression in embryos exposed to this compound (Fig. 3E, Table S1). The effective concentrations that achieved 1.5-fold increase over control (EC1.5) varied from sub-micromolar to millimolar scale (Fig. 3A–E; Table S2), in good dosage agreement with impaired motility, particularly for the compounds which have effects on *hspb11* expression already well below lethality as the apical endpoint (Fig. S1; Table S2).

**Figure 4:**
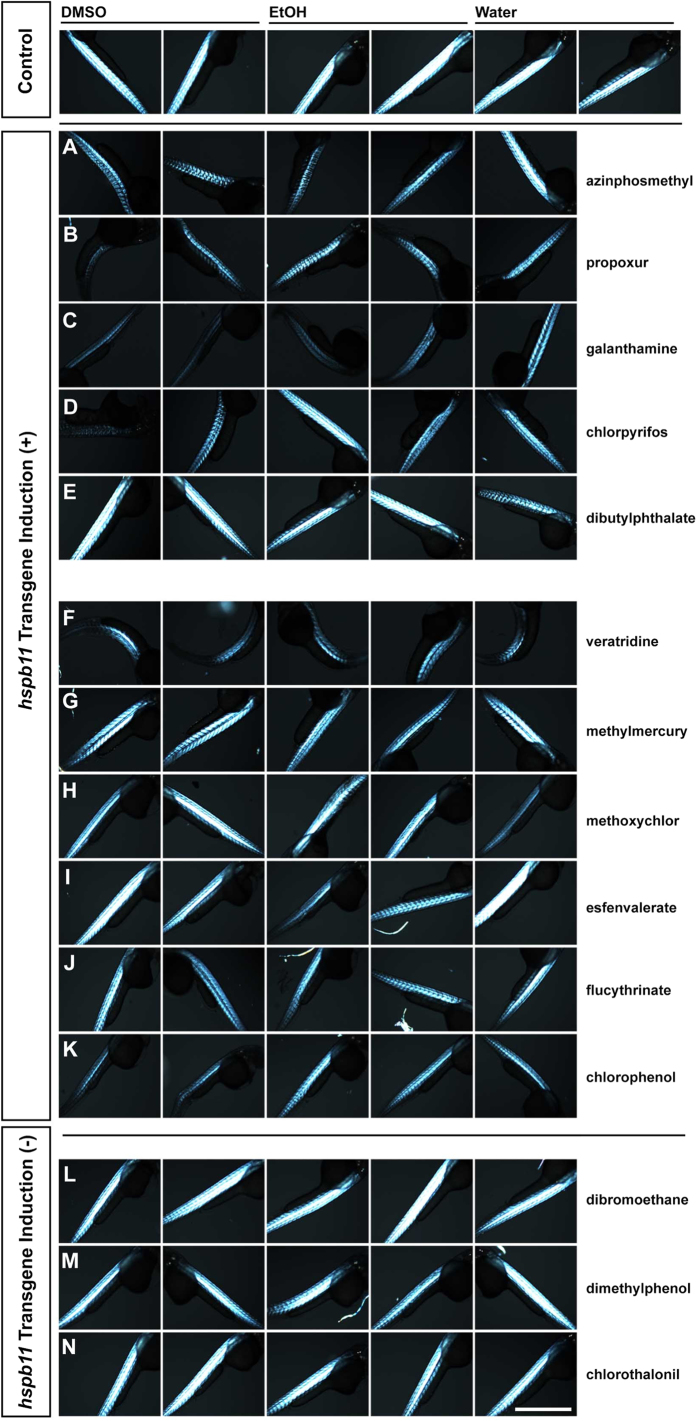
Reduced muscle integrity in correlation to *hspb11* transgene up-regulation. Birefringence images from the trunk region of wildtype embryos, treated during 9–48 hpf with either azinphosmethyl (**A**, 3 μM), propoxur (**B**, 720 μM), galanthamine (**C**, 1 mM), chlorpyrifos (**D**, 20 μM), dibutylphthalate (**E**, 2.69 μM), veratridine (**F**, 120 μM), methylmercury (**G**, 0.119 μM), methoxychlor (**H**, 2.3 μM), esfenvalerate (**I**, 0.191 µM), flucythrinate (**J**, 0.055 µM), chlorophenol (**K**, 389 μM), dibromoethane (**L**, 2.1 mM), dimethylphenol (**M**, 327 μM) and chlorothalonil (**N**, 0.4 μM). Five representative examples are shown for each chemical. Scale bar: 500 μm.

Ion channels are often targets of medicines and toxicants. Normal motor function requires precise control of voltage-gated Na^+^ channel function for the transformation of focal membrane depolarization into a fibre-wide action potential and the ensuing release of intracellular calcium ions for actin-myosin interaction. We examined the effects of voltage-gated Na^+^ channel activators (veratridine, flucytrinate and esfenvalerate), of methylmercury that causes up-regulation of intracellular calcium levels, and of other channel modulators (methoxychlor and chlorophenol) (Fig. 3F–K). For all channel modulators, we observed dose-dependent up-regulation of *hspb11* transgene expression, again in good dosage agreement with impaired motility (Fig. S1; Table S2). Channel modulator treatment led to reduced muscle integrity as well (Fig. 4F–K). Thus, *hspb11* transgene up-regulation was again found to correlate with motor dysfunction, as we observed with AChE inhibitors.

To confirm the specificity of the *hspb11* transgene response as an indicator of motor dysfunction, we also tested some other compounds with no previously reported effects on muscle birefringence in treated embryos: dibromoethane (oxidative phosphorylation disruptor), dimethylphenol (disruptor of ATPase activity) and chlorothalonil (thiol-reactive) (Fig. 4L–N). The *hspb11* transgene levels were not significantly different from solvent control after treatments with the three compounds (Fig. 3L–N, one-way ANOVA *p *> 0.001). Importantly, all three chemicals induced impaired motility (Fig. S1L–N), while none of them showed reduction in muscle integrity (Fig. 4L–N). Given the mode of action of these compounds (interference with energy metabolism, Table S1), the reduction of motility could be related to the depletion of the cellular energy storage and subsequent inability to conduct muscle contraction. In contrast, AChE inhibitors or sodium channel activators would lead to an over-excitation of muscles and impairment of motility due to increased muscle activity. The far lower *hspb11* EC1.5 concentrations in comparison to corresponding LC50 values observed with propoxur (37.8-fold lower) and chlorpyrifos (19.5-fold) further indicate the specificity of *hspb11* transgene response (Table S2). Taken together, the *hspb11* transgene system detected the presence of chemicals of a wide range of structures which cause impaired muscle integrity.

### *hspb11* reporter induction accompanies muscle hyperactivity

**Figure 5:**
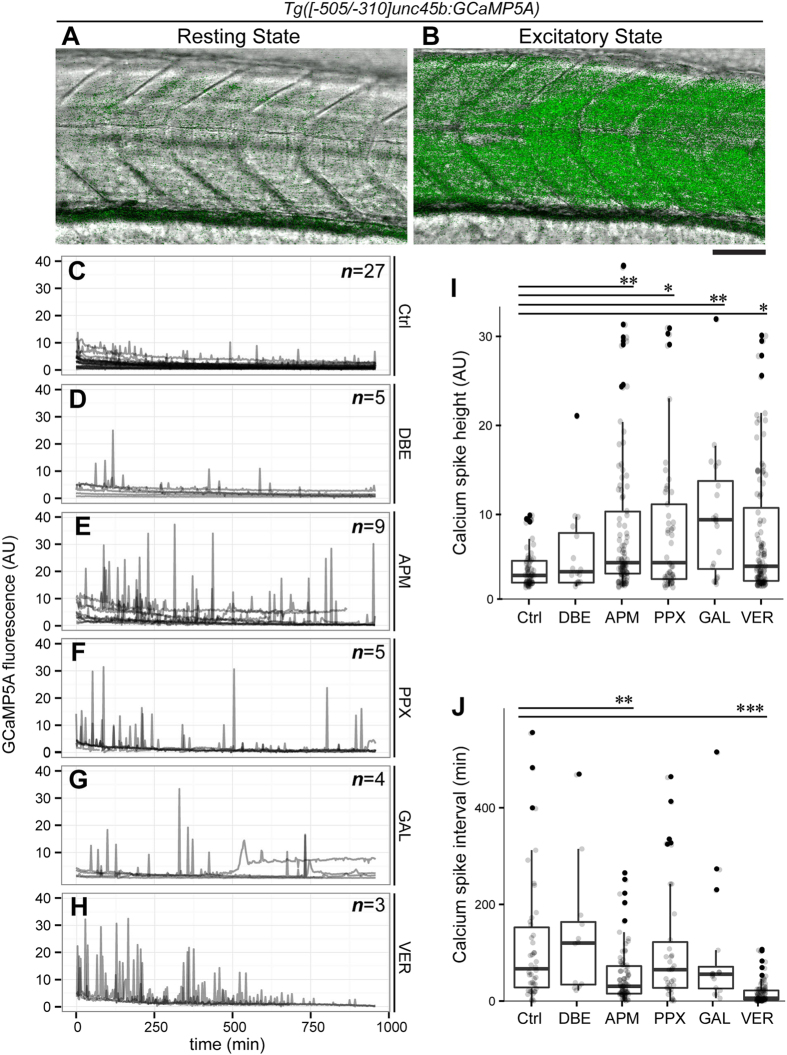
Up-regulation of *hspb11* transgene accompanies muscle hyperactivity. (**A,B**) Lateral views of the trunk region of a muscle-specific calcium sensor line *Tg*(*[-505/-310]unc45b:GCaMP5A*) at 72 hpf. The fluorescent channel images for calcium (green) are merged with the bright field transmission images (gray) for non-contractile resting state (**A**) and excitatory state (**B**), exhibiting low and high fluorescent reporter levels, respectively. Anterior side is oriented to the left. Scale bar: 50 μm. (**C–H**) Profiles of calcium levels in the trunk skeletal muscle from individual embryos treated with either DMSO solvent control (Ctrl, **C**), dibromoethane (DBE, **D**), azinphosmethyl (APM, **E**), propoxur (PPX, **F**), galanthamine (GAL, **G**) or veratridine (VER, **H**) plotted as a function of time (temporal resolution: 2.5 minutes). The number of examined embryos is shown at the top-right of each panel. (**I–J**) Calcium profiles shown in the panels (**C–H**) were analyzed for the height of individual calcium spikes (**I**) and the temporal interval of two adjacent calcium spikes (**J**) and presented in whisker-box charts with individual data shown as gray dots. Black dots represent outliers. (**I**) One-way ANOVA showed significant differences of calcium spike height (*F*[5,315]* *= 4.1464, *p *= 0.00116) between solvent control (*n *= 55 spikes), dibromoethane (*n *= 16 spikes), azinphosmethyl (*n *= 89 spikes), propoxur (*n *= 45 spikes), galanthamine (*n *= 20 spikes), veratridine (*n *= 96 spikes). Significant differences over the solvent control were found with azinphosmethyl (TukeyHSD test from here onward, **p = 0.002698), propoxur (*p = 0.01845), galanthamine (**p = 0.009827) and veratridine (*p = 0.01391) treatments, while dibromoethane showed no difference over the control treatment (*p *= 0.9317). (**J**) Significant differences of calcium spike intervals were detected (one-way ANOVA, *F*[5,256]* *= 12.422, *p *= 8.405 × 10^−11^) between solvent control (*n *= 42 intervals), dibromoethane (*n *= 12 intervals), azinphosmethyl (*n *= 75 intervals), propoxur (*n *= 34 intervals), galanthamine (*n *= 16 intervals), veratridine (*n *= 83 intervals). Azinphosmethyl (TukeyHSD test from here onward, **p = 0.003285) and veratridine (***p = 1.0 × 10^−7^) treatments significantly shortened the interval of calcium spikes, while dibromoethane (*p *= 0.9744) galanthamine (*p *= 0.9895) and propoxur (*p *= 0.9999) were comparable with the control treatment.

To correlate muscle physiology more directly with *hspb11* up-regulation, we established a muscle specific Ca^2+^ biosensor line *Tg*(*[-505/-310]unc45b:GCaMP5A*). We put the GCaMP5a cassette^29^ under the control of regulatory elements of the *unc45b* gene (Etard *et al.*, unpublished), which confer skeletal muscle-specific reporter gene expression (Fig. 5A–B). We exposed embryos at 72 hpf with solvent control, with chemicals that induce *hspb11* transgene expression (azinphosmethyl, propoxur, galanthamine and veratridine; Fig. 3A–C,F) and with dibromoethane, which does not induce *hspb11* transgene expression (Fig. 3L), and measured Ca^2+^ fluctuations under a confocal microscope for 16 hours (Fig. 5C–H). To quantify the effects of chemicals on the dynamics of Ca^2+^ fluctuations, we compared the height of the Ca^2+^ peaks (Fig. 5I) as well as the time intervals between two adjacent Ca^2+^ peaks (Fig. 5J). In comparison with the control treatment, exposure to azinphosmethyl and galanthamine led to a significant increase in the height of calcium spikes (**p < 0.01, Fig. 5I). Similar effects were observed with propoxur and veratridine (*p < 0.05, Fig. 5I). In terms of the frequency of the calcium spikes, significantly reduced time intervals were observed with azinphosmethyl and veratridine in comparison to the control (**p < 0.01, Fig. 5J). Dibromoethane, which does not induce *hspb11* transgene expression (Fig. 3L), showed no effects on the calcium dynamics compared to the control group (Fig. 5I–J). These increases of calcium spike intensity and frequency are hallmarks of muscle hyperactivity. Thus, we identified induction of muscle hyperactivity as a common feature of chemicals that up-regulate the *hspb11* transgene.

### Chemically induced up-regulation of *hspb11* transgene requires muscle hyperactivity

The *hspb11* transgene up-regulation occurs concomitantly with muscle hyperactivity (Figs 3 and 5). This raises the question whether it is the hyperactivity *per se* that causes the up-regulation of *hspb11* transgene expression, irrespective of the state of muscle integrity. To address this question, we examined whether up-regulation of *hspb11* transgene expression is suppressed when muscle contraction is abolished by an anaesthetic.

**Figure 6:**
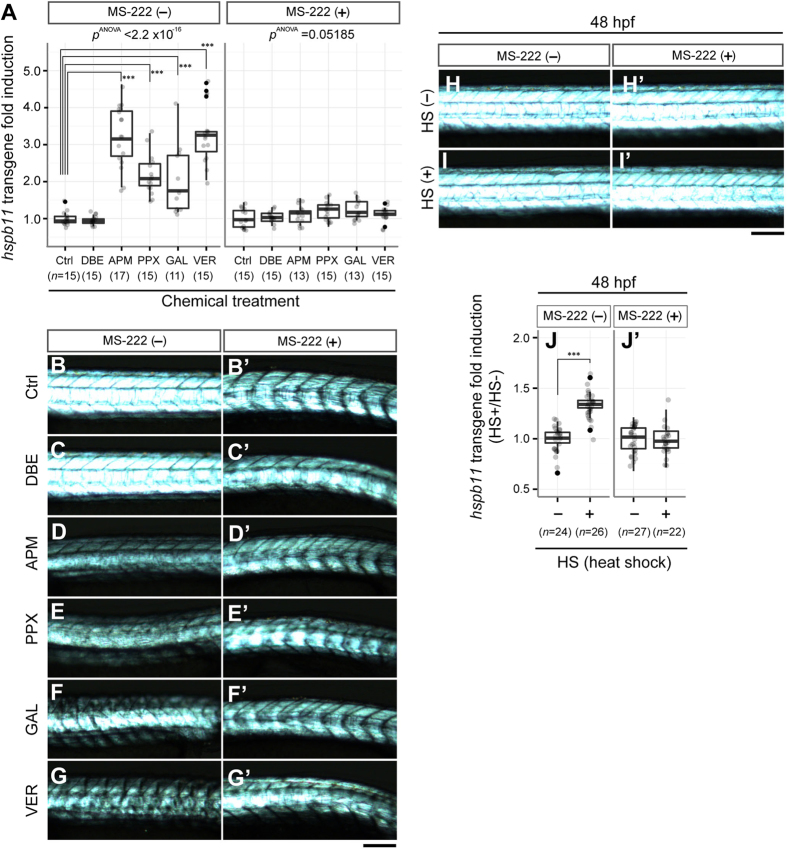
Chemical–or heat shock-induced up-regulation of *hspb11* transgene expression is caused by muscle hyperactivity. (**A**) Chemical induction of *hspb11* transgene expression. *TgBAC*(*hspb11:GFP*) embryos were treated with azinphosmethyl (APM), propoxur (PPX), galanthamine (GAL) or veratridine (VER) during 9–48 hpf with negative controls of DMSO control (Ctrl) and dibromoethane (DBE), either in the absence or in the presence of MS-222. The *hspb11* GFP reporter intensity in the trunk region is expressed as fold induction over DMSO control. In the absence of MS-222, a significant increase of *hspb11* transgene expression in comparison to the DMSO control was observed (one-way ANOVA *F*[5, 82]* *= 40.732, *p *= 2.2 × 10^−16^) with azinphosmethyl (TukeyHSD, ***p = 0.0000000), propoxur (***p = 0.0000165), galanthamine (***p = 0.0007428), and veratridine (***p = 0.0000000). However, this was not the case in the presence of MS-222 (*F*[5, 80]* *= 2.3075, *p *= 0.05185). Sample size is given in parenthesis as the number of embryos. (**B–G’**) Birefringence images of representative embryos from the experiment shown in panel (**A**) (lateral views, anterior left). (**H–I’**) Birefringence images of representative embryos from the experiment quantified in (**J,J’**) at 48 hpf treated with a heat shock (HS) of 1 hour at 37 °C in the absence (**J**) or the presence (**J’**) of MS-222. (**J–J’**) HS-mediated up-regulation of *hspb11* transgene expression. *TgBAC*(*hspb11:GFP*) embryos at 48 hpf were treated either with HS (37 °C, 1 hour) or left untreated, and HS effects on *hspb11* transgene expression were evaluated either in the absence or in the presence of MS-222. In the absence of MS-222, HS induced a significant increase of *hspb11* transgene expression (Welch two-sample *t*-test; *t *= −11.2483, ***p = 1.036 × 10^−14^). In contrast, no significant increase was observed in the presence of MS-222 (Welch two-sample *t*-test; *t *= 0.3744, *p *= 0.7098). Sample size is given in parenthesis as the number of embryos. Scale bars: 200 μm.

For chemical induction of *hspb11* transgene expression, we used azinphosmethyl (3 μM), propoxur (720 μM), galanthamine (1 mM) and veratridine (120 μM), with DMSO (0.05%) and dibromoethane (2.1 mM) as negative controls (Fig. 3). In the absence of the anaesthetic MS-222 (tricaine methanesulfonate, 643 μM), we observed significant up-regulation of *hspb11* transgene in treatments with all four compounds in comparison to the respective solvent controls (Fig. 6A; ***p < 0.001, Tukey honest significant difference (HSD) test). Co-exposure with MS-222, however, abolished the up-regulation of *hspb11* transgene expression by these compounds (Fig. 6A, *p *= 0.05185 one-way ANOVA), suggesting that muscle contraction is required for the up-regulation of the *hspb11* transgene.

Next, we examined whether there is still a correlation between *hspb11* up-regulation and muscle disintegration when muscle contraction is absent. As reported previously^30^, we observed reduced muscle integrity even with solvent-control embryos when they were raised in the presence of MS-222 (Fig. 6B’), reflecting the adverse effects of MS-222 on the development of the somite (data not shown). Although we observed similarly reduced muscle integrity after exposure to MS-222 with or without chemicals (Fig. 6B’–G’), we did not observe up-regulation of the *hspb11* transgene with any of the treatments (Fig. 6A). Thus, in the absence of muscle contraction *hspb11* transgene levels do not correlate with reduced muscle integrity, indicating again the requirement of muscle activity for the up-regulation of the *hspb11* transgene.

### Up-regulation of *hspb11* transgene expression by heat shock is also caused by muscle hyperactivity

We observed that *hspb11* transgene expression appears to be up-regulated by muscle hyperactivity, but not necessarily by muscle disintegration (Fig. 6A–G’). Thus, conditions should exist where *hspb11* transgene expression is up-regulated in the absence of muscular disintegration. To test this hypothesis, we selected heat shock as a means for fine controlling *hspb11* transgene induction and explored heat shock conditions that do not cause muscle disintegration. A heat shock at 37 °C for 1 hour did not reduce muscle integrity of treated embryos at 48 hpf; 97% embryos (n = 31) showed normal birefringence (Fig. 6I), comparable to the control group without heat shock (n* *= 30 [97%], Fig. 6H). We applied this heat shock condition to *TgBAC*(*hspb11:GFP*) embryos at 48 hpf and measured *hspb11* transgene levels at 4–5 hours after the heat shock.

In the absence of MS-222, the heat shock treatment significantly increased *hspb11* transgene levels at both 24 hpf (data not shown) and 48 hpf (Fig. 6J; ***p < 0.001, Welch two sample *t*-test). In the presence of MS-222 during the 1-hour period of heat shock, however, up-regulation of *hspb11* transgene expression was eliminated at both stages of development (Fig. 6J’, *p *= 0.7098). This indicates that *hspb11* transgene expression was up-regulated not by the heat itself, but rather by muscle hyperactivity during the heat shock. Accordingly, we observed increased motility during heat shock treatment in the absence of MS-222 (data not shown). Importantly, we did not observe muscle disintegration in the presence of MS-222 for 1-hour with or without heat shock; for each group *n *= 29/30 [97%] embryos showed normal birefringence. This result supports our hypothesis that it is not reduced muscle integrity but rather its hyperactivity that causes the up-regulation of *hspb11* transgene expression.

## Discussion

We report here that we established a transgenic zebrafish line *TgBAC*(*hspb11:GFP*) as a tool for monitoring chemicals that induce muscle hyperactivity. This transgenic line does not require time-lapse analysis for detecting muscle hyperactivity, as it converts a dynamic process into a quantifiable single readout. Because it does not assume any particular class of chemical compounds, it provides robust detection power. Indeed, chemicals that up-regulate *hspb11* transgene expression show a wide variety of chemical structures (Fig. 3). This transgenic line detects chemicals that modify normal neuromuscular transmission between motor neurons and muscle fibres. Therefore, it may not detect chemicals that interfere with higher motor function without inducing muscle hyperactivity, which is integrated with sensory system and/or cognitive function (e.g. locomotion, social behaviour).

One criticism against using heat shock proteins for any biosensor is the potential of a high false positive rate through their responsiveness to a wide range of stresses, such as heat shock or ROS exposure. However, our results show that the *hspb11* transgene specifically responds to muscle hyperactivity; a heat shock does not induce its expression if muscle activity is inhibited in parallel (Fig. 6). In zebrafish, there are 13 sHSPs, and five of them (*hspb1*, *hspb7*, *hspb8*, *hspb9* and *hspb11*) are expressed in the muscle, where they show induced expression upon heat shock^31^,^32^. Our finding that *hspb11* transgene expression responds to muscle hyperactivity rather than directly to the heat shock raises the possibility that these “heat shock-inducible” sHSPs expressed in the muscle may also primarily respond to muscle hyperactivity. This unique hyperactivity-dependent *hspb11* transgene induction precludes false-positive responses arising from general stresses associated with sample handling during a chemical screen. The high signal-to-noise ratio of up to 3-fold of GFP intensity after toxicant exposure is another beneficial feature of our *hspb11*-based system.

If *hspb11* transgene expression is regulated in a muscle activity-dependent manner, how should one interpret its expression in the notochord? Is our strategy to combine both muscular and notochordal *hspb11* transgene expression inappropriate? To address this issue, we measured *hspb11* transgene levels separately for both single tissues after chemical treatment (Fig. S3). In the absence of MS-222, azinphosmethyl, propoxur and veratridine treatment up-regulated *hspb11* transgene in both single tissues more than 1.5-fold over negative controls (DMSO and dibromoethane). Interestingly, the presence of MS-222 eliminated the up-regulation of *hspb11* transgene both in the muscle and the notochord (Fig. S3). Thus, both muscular and notochordal *hspb11* transgene are regulated by a similar mechanism. The notochord of amphioxus, a non-vertebrate chordate, is contractile and takes part in body movements together with myotomal muscles^33^. Thus, the vertebrate notochord may retain molecules common to the muscle, even though it is no longer contractile. Functional studies on the role of *hspb11* in the notochord are pending.

The *TgBAC*(*hspb11:GFP*) zebrafish biosensor described here provides an unique tool for detecting chemicals that induce muscle hyperactivity, thereby interfering with motor function. It provides a quantitative measure for muscle hyperactivity without time lapse analysis, featuring high signal-to-noise ratio, sensitivity and specificity. A simple readout potentiates its use for increased throughput. Alternatively, the *hspb11* sensor could easily be combined with the Ca^2+^ sensor by simply crossing differently coloured transgenic reporters into one line. Automated measurements of these fluorescent readouts combined with the measurements of altered behavioural effects such as the motor response (e.g. vibration stimulated^34^), the photomotor response^35^ or heart beat^36^ will provide a multi-parameter assessment of the toxicological impact of compounds. This set-up will provide, in a single assay, insights into toxicological effects far beyond what can currently be achieved in cell culture or *in vitro* organ systems. As all these measurements can be carried out with zebrafish embryos, these assays will contribute to the principle of the 3Rs for ethical use of animals in testing (Replacement, Reduction and Refinement^37^) and at the same time satisfy the demands of the REACH programme^38^.

## Methods

### Ethics statement

All zebrafish husbandry and experimental procedures were performed in accordance with the German animal protection standards and were approved by the Government of Baden-Württemberg, Regierungspräsidium Karlsruhe, Germany (Aktenzeichen 35-9185.64).

### Fish Maintenance

Wild-type zebrafish ABO is an inbred line initially derived from an intercross between the AB and wtOX line. The wild-type zebrafish strains ABO, and transgenic zebrafish lines *TgBAC*(*hspb11:GFP*) and *Tg*(*-[505–310]unc45b:GCaMP5A*) were maintained on a 14 hr/10 hr light-dark cycle at 28.5 °C in a recirculation systems (Schwarz Ltd., Göttingen, Germany) and fed with commercial food and in-house hatched brine shrimp as described^39^. Embryos were reared in embryo medium and staged according to Kimmel *et al*^40^.

### Generation of *TgBAC(hspb11:GFP)* and *Tg(-[505/-310]unc45b:GCaMP5A)* transgenic fish

The zebrafish BAC clone for *hspb11* (DKEY-30K6 (ID:1132288); Sequence ID: AL844141.7; 257634 bp) was purchased from Source BioScience (Berlin, Germany). The modified target vector with homologous arms of *hspb11*, enhanced green fluorescent protein (GFP) and kanamycin resistance sequences was generated as previously described^22^. The subsequent insertion of the iTol2-amp cassette into the modified BAC DNA for Tol2 transposase mediated facilitated integration of the construct into the zebrafish genome was performed following Suster *et al*.^24^. The modification of BAC DNA was confirmed by PCR^23^, and the clones positive for GFP and Tol2 arms were selected for micro-injection with Tol2 transposase mRNA. Stable transgenic lines were identified by GFP expression in the out-crossed offspring from G0 founder fish (F1). Two positive F1 families derived from independent G0 founders were further in-crossed to obtain homozygous transgene carriers (F2). For the experiments, F2 homozygous carriers were out-crossed to obtain identical genetic population with heterozygous transgene expression. For the generation of the muscle specific calcium sensor line *Tg*(*[-505/-310]unc45b:GCaMP5A*), the 5′-untranslated region (from the position −505 to −310 relative to the translation start site, designated as [−505/−310]) of the gene coding for myosin chaperon *unc45b* was used to drive the calcium sensor GCaMP5A cassette in a muscle specific manner. The expression vector [*−505/−310*]*unc45b*-GCaMP5A flanked by Tol2-sites was created using the Tol2-kit^41^. The expression vector (concentration 5 ng/μl) was co-injected with Tol2 transposase mRNA (10 ng/μl) for genomic integration. The injected embryos (G0) were raised for identification of insertions.

### Chemicals

The chemicals used in this study were purchased from Sigma-Aldrich: azinphosmethyl (*O*,*O*-dimethyl *S*-[(4-oxo-1,2,3-benzotriazin-3(4*H*)-yl)methyl]dithiophosphate; CAS# 86-50-0; dissolved in dimethylsulfoxide [DMSO]), propoxur (2-isopropoxyphenyl *N*-methylcarbamate; CAS# 114-26-1; dissolved in H_2_O), galanthamine ((4a*S*,6*R*,8a*S*)– 5,6,9,10,11,12– hexahydro– 3-methoxy –11-methy l–4a*H* –[1]benzofuro[3a,3,2-*ef*] [2] benzazepin –6-ol; CAS# 357-70-0, dissolved in DMSO), chlorpyrifos (*O*,*O*-diethyl *O*-3,5,6-trichloropyridin-2-yl phosphorothioate; CAS# 2921-88-2, dissolved in ethyl alcohol [EtOH]), chlorophenol (4-chlorophenol; CAS# 106-48-9, dissolved in H_2_O), dibutylphthalate (di-*n*-butylphthalate; CAS# 84-74-2, dissolved in DMSO), veratridine ((3β,4β,16β)-4,12,14,16,17,20-hexahydroxy-4,9-epoxycevan-3yl 3,4-dimethoxybenzoate 3-veratroylveracevine; CAS# 71-62-5, dissolved in DMSO), flucythrinate (cyano(3-phenoxyphenyl)methyl 2-[4-(difluoromethoxy)phenyl]-3-methylbutanoate; CAS# 70124-77-5, dissolved in EtOH), methoxychlor (1,1,1-trichloro-2,2-bis(4-methoxyphenyl)ethane; CAS# 72-43-5, dissolved in EtOH), esfenvalerate ((*S*)-cyano (3-phenoxyphenyl) methyl-(*S*)-4-chloro-alpha-(1-methylethyl); CAS# 66230-04-4, dissolved in EtOH), methylmercury (methylmercury(II) chloride; CAS# 115-09-3, dissolved in H_2_O), dibromoethane (1,2−dibromoethane, CAS# 106-93-4, dissolved in H_2_O), dimethylphenol (2,4-dimethylphenol, CAS# 105-67-9, dissolved in H_2_O), chlorothalonil (2,4,5,6-tetrachloroisophthalonitrile; CAS# 1897-45-6, dissolved in DMSO) and MS-222 (tricaine methanesulfonate; ethyl 3-aminobenzoate methanesulfonate; CAS# 886-86-2, dissolved in H_2_O).

### Chemical treatment

Embryos were collected and raised in embryo medium^39^ until 6 hpf (hours post fertilization). The fertilized embryos were selected and arranged in a 6-well polystyrene plate. Exposure of embryos to chemicals with desired concentrations was performed from 9–48 hpf in embryo medium with 0.003% phenylthiourea (*N*-phenylthiourea; PTU) for preventing the formation of pigmentation. In dose response experiments for chemicals, embryos were treated in various concentrations between 0.01 μM to 3 mM. Final solvent (DMSO and EtOH) concentrations were never higher than 0.1%. Embryos were manually dechorionated at 48 hpf before imaging. The experiment was performed in three biological repeats with at least 10 embryos at each dose from a series of concentrations.

### Heat shock treatment

For the heat shock experiment, *TgBAC*(*hspb11:GFP*) transgenic embryos at 48 hpf were divided into 4 groups of 20–30 embryos each in the 2 ml tubes, filled with 1 ml of fish water. In two of the tubes the anaesthetic agent MS-222 was added at 643 μM, while the other two were kept as MS-222 negative. The temperature of the water bath was set to 37 °C and the heat shock was given for 1-hour to two groups of embryos, one with MS-222 and another without. The other pair of two groups, with or without MS-222, was kept at room temperature. After 4–5 hours post heat shock, images were taken under a compound microscope to quantify *hspb11* transgene levels and to investigate the effects on muscle integrity by examining birefringence.

### Scoring for lethality and abnormal motility

The embryos were examined for motility and lethality at 48 hpf. Abnormal motility includes continuous twitch or shivering movements in the absence of touch stimuli, and lack of escape behaviour upon touch stimuli with the tip of a hair at the trunk. The experiment was performed in three biological repeats with at least 10 embryos each. Lethality was scored by lack of heart beat and coagulated bodies in a separate set of three experiments with at least 12 embryos each.

### Detection of birefringence

Muscle integrity was observed under the compound microscope DM5000 with a 5 ×objective using polarized light^42^. Control and treated embryos were rotated in a way that a maximum amount of light could pass through the muscle fibres. Exposure time was set to 5.63 ms, gamma was 1.13 and gain was 4.0.

### *In situ* gene expression analysis and histology

Whole-mount *in situ* hybridisation was performed as described^43^, with minor modification. Briefly, fixed embryos were treated with chilled acetone at −20 °C for 7 minutes to enhance tissue penetration of digoxygenin-labelled anti-sense ribonucleic acid probes. Hybridisation was performed at 65 °C in the presence of 1 mM EDTA to block endogenous alkaline phosphatase activity. After colour development with bromo-4-chloro-3-indolyl phosphate (BCIP, Roche) and nitroblue tetrazolium chloride (NBT, Roche), embryos were embedded into epoxy resins and 5 μm thick transverse sections were cut at the level of trunk as described^44^.

### Algorithmic Parameters and Statistical Analysis

The dechorionated embryos were mounted in 3% methyl cellulose with dorsal up. Images were taken at 48 hpf using a Leica compound microscope DM5000 under a 10 × objective. Exposure time was set to 880 ms for optimal visualization. The remaining parameters, i.e., gamma, saturation and gain were set to 1:18, 1:5 and 4.6, respectively. For each embryo, a single RGB image of the *hspb11* reporter GFP signal was captured at a resolution of 1392 × 1040 pixels (px) with a physical spacing of 1 px* *= 0.32 μm. All acquired images were converted into a grey scale for further processing as described^25^, with minor parameter adjustments to adapt the processing steps to the different image contents (Gaussian-smoothing with a standard deviation of σ* *= 10 and region of interest extraction with a radius of r* *= 350 px). Global statistical values such as minimum, maximum, median, mean and standard deviation of intensity values were extracted from the linearized and cropped original image. All algorithms were implemented in MATLAB as an extension package to the open-source MATLAB toolbox Gait-CAD^45^. To evaluate whether there is an effect of a test chemical on the *hspb11* reporter level, the differences of the *hspb11* reporter level among doses including solvent control were examined by one-way ANOVA with a significance threshold of *p *< 0.001. Chemicals with significant effects were modelled for their concentration dependent responses using a Gaussian 4-parameter logarithmic model after normalising the *hspb11* transgene levels by the mean of the control (equation 1)^46^:


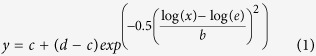


The coefficient *c* was set to 1 due to the prior normalisation. *b*, *d* and *e* were fitted using the program jmp 11.1.1. (SAS, Böblingen, Germany). Subsequently, the concentrations (*x*) leading to a 1.5 (*y*) induction were calculated (EC1.5). The effect level of 1.5-fold was selected based on the average control variability. In case of methylmercury, dibutylphthalate, methoxychlor and 4-chlorophenol, no Gaussian like concentration-response was observed and the 1.5-fold induction levels were estimated from a linear regression model^47^.

### Confocal time lapse analysis

2-dpf transgenic embryos *Tg*(*[-505/-310]unc45b:GCaMP5A*) were embedded into 0.5% low-melting agarose prepared with 50 mM HEPES pH 7.0/ 5 mM NaCl/ 0.17 mM KCl/ 0.33 mM CaCl_2_/ 0.33 mM MgSO_4._ Each chemical treatment (dibromoethane at 2.1 mM, azinphosmethyl at 1 μM, propoxur at 720 μM, galanthamine at 1 mM and veratridine at 120 μM) was accompanied by solvent control (0.5% DMSO) in a home-made imaging chamber with two isolated slots. No anaesthetic chemical was added. A Leica TCS SP5 upright confocal microscope was used in resonant scanning mode (bidirectional scanning at 8000 Hz) to achieve a time resolution of 2.5 minutes with a 2 μm z-step size for up to 12 embryos for 16 hours. A HCX PL APO lambda blue 20x NA0.70 IMM UV objective was used with glycerol as immersion medium. Temperature control was set to 28 °C. Data were processed with ImageJ/Fiji^48^.

## Additional Information

**How to cite this article**: Shahid, M. *et al.* Zebrafish biosensor for toxicant induced muscle hyperactivity. *Sci. Rep.* **6**, 23768; doi: 10.1038/srep23768 (2016).

## Supplementary Material

Supplementary Information

Supplementary Video 1

Supplementary Video 2

Supplementary Video 3
