# Supplementary material for: Zebrafish biosensor for toxicant induced muscle hyperactivity
Source: Sci Rep. 2016 Mar 31;6:23768. doi: 10.1038/srep23768 (PMC4815012; doi:10.1038/srep23768)
Supplement: Supplementary Information [file srep23768-s1.pdf]

Zebrafish biosensor for toxicant induced muscle hyperactivity

Maryam Shahid[1,5], Masanari Takamiya[1,5], Johannes Stegmaier[3,5], Volker Middel[1], Marion Gradl[1], Nils Klüver[4], Ralf Mikut[3], Thomas Dickmeis[1], Stefan Scholz[4], Sepand Rastegar[1], Lixin Yang[1,2*] and Uwe Strähle[1,*]

Affiliations:

1. Institute of Toxicology and Genetics, Karlsruhe Institute of Technology (KIT), Postfach 3640, 76021 Karlsruhe and university of Heidelberg, Germany.
2. State Key Laboratory of Environmental Criteria and Risk Assessment, Chinese Research Academy of Environmental Sciences, 100012, Beijing, China.
3. Institute for Applied Computer Sciences, Karlsruhe Institute of Technology (KIT), Postfach 3640, 76021 Karlsruhe, Germany.
4. Department of Bioanalytical Ecotoxicology, UFZ - Helmholtz Centre for Environmental Research, Leipzig, Germany.
5. These authors contributed equally.

*Correspondence to Lixin Yang and Uwe Strähle

Supplementary information

Figure S1

Motility defects of embryos treated with various chemicals.

Effects of individual tested chemicals on motility were examined at various doses. AChE inhibitors: (A) azinphosmethyl, (B) propoxur, (C) galanthamine, (D) chlorpyrifos and (E) dibutylphthalate. Channel modulators: (F) veratridine, (G) methylmercury, (H) methoxychlor, and (I) chlorophenol and other toxicants: (J) dibromoethane, (K) dimethylphenol and (L) chlorothalonil. The embryos that failed to swim away two times out of three touch trials were categorized for impaired motility. The percentage of embryos with impaired motility is presented as a column for each dose (µM). The number in ratio format shown above each column represents the number of embryos with impaired motility over total examined embryos (impaired/total embryos). Fisher's exact test was performed between solvent control (Ctrl) and each examined dose to evaluate the statistical significance of the chemical exposures on motility. Results were adjusted with Bonferroni correction for multiple

comparisons and designated as * for $p<0.05$, **for $p<0.01$ and *** for $p<0.001$. Hill slope modelling for EC50 calculation are shown when applicable.

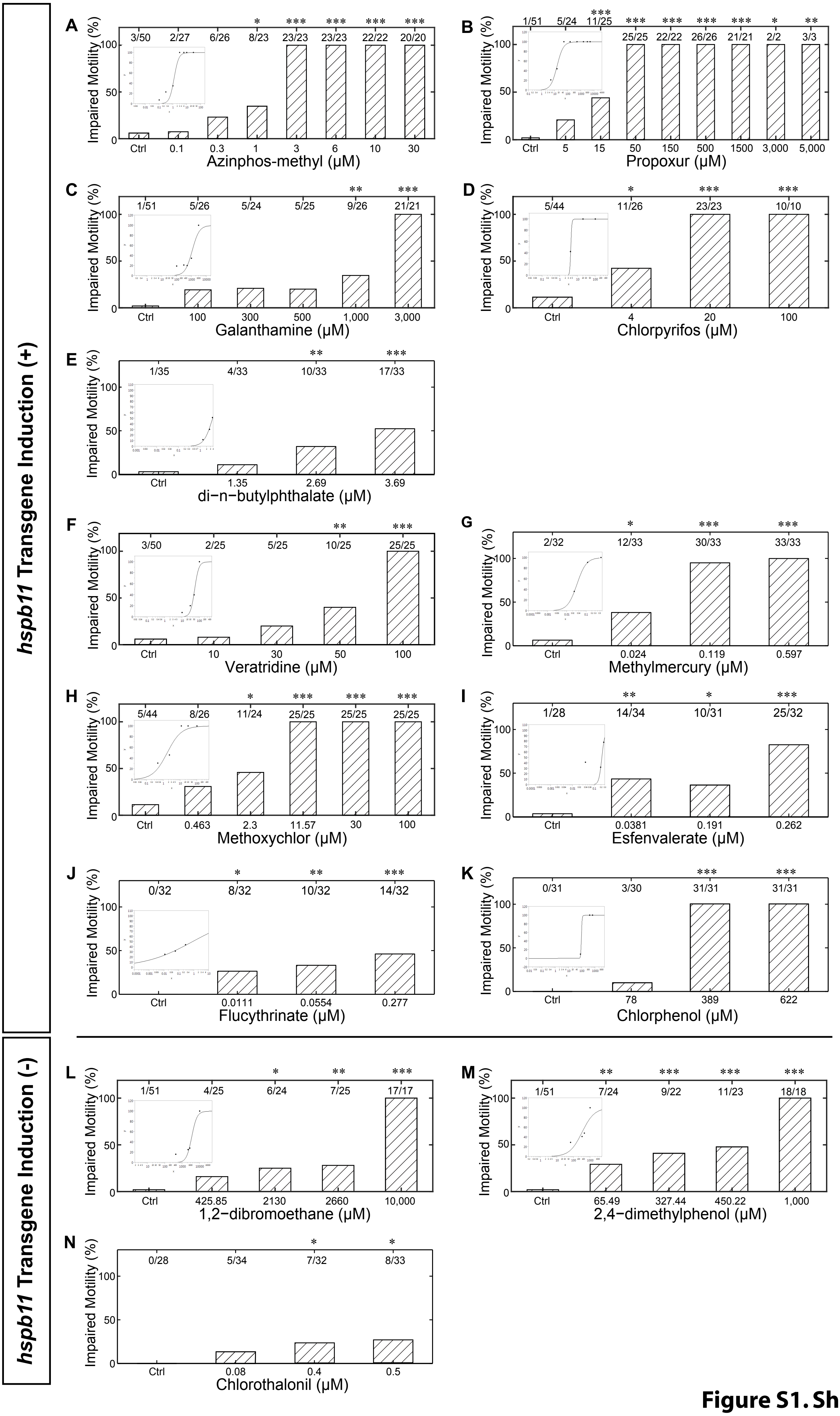

Figure S1. Shahid *et al.*

Figure S2

Lethality among embryos treated with various chemicals.

Wildtype embryos were treated with various chemicals during 9-48 hpf and examined for the lethality of the compounds. The selection of dead embryos was based on the absence of heart beat and coagulated bodies. AChE inhibitors: (A) azinphosmethyl, (B) propoxur, (C) galanthamine, (D) chlorpyrifos and (E) dibutylphthalate. Channel modulators: (F) veratridine, (G) methylmercury, (H) methoxychlor, and (I) chlorophenol and other toxicants: (J) dibromoethane, (K) dimethylphenol and (L) chlorothalonil. The percentage of dead embryos is presented as a column for each dose (µM). The number in ratio format shown above each column represents the number of dead embryos over total examined embryos (dead/total embryos). Fisher's exact test was performed between solvent control (Ctrl) and each examined dose to evaluate the statistical significance of the chemical exposures on the lethality. Results were adjusted with Bonferroni correction for multiple comparisons and designated as * for $p<0.05$, ** for $p<0.01$ and *** for $p<0.001$. Hill slope modelling for LC50 calculation are shown when applicable.

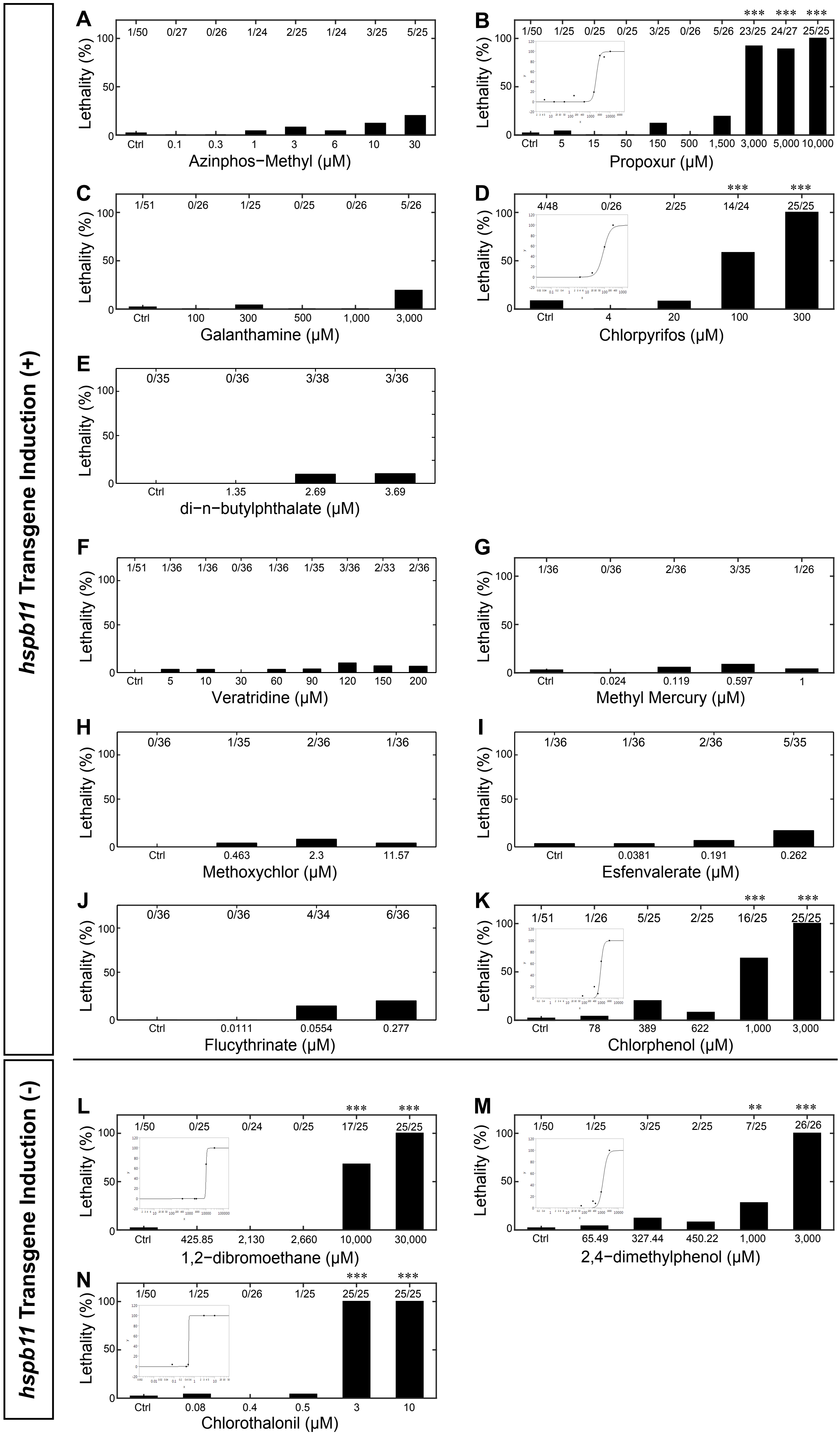

Figure S2. Shahid *et al.*

Figure S3

Comparison of chemically-induced hspb11 transgene response in the muscle and in the notochord

(A) Embryos from *TgBAC(hspb11:GFP)* were treated with azinphosmethyl (APM), propoxur (PPX), or veratridine (VER) during 9-48 hpf with two negative controls, a DMSO solvent control (Ctrl) and dibromoethane (DBE), in the absence of MS-222 [MS-222(-)] and in the presence of MS-222 [MS-222(+)]. The *hspb11* transgene intensity at the trunk region is separately measured for the muscle and the notochord. The intensity of GFP reporter fluorescence is expressed as fold induction over control. Two-way ANOVA revealed significant main effects of tissue (muscle or notochord; $F[1,446]=4.1917$, $p=0.04121$) and MS-222 ($F[1,446]=126.2648$, $p<2 \times 10^{-16}$) with no significant interaction between tissue and MS-222 treatments ($F[1,446]=1.3640$, $p=0.24347$). Significant induction of *hspb11* reporter by chemicals was observed in all cases independently from tissues and MS-222 treatment, as indicated in each panel by one-way ANOVA *p*-values. However, biologically meaningful effects by chemicals were observed only in the absence of MS-222, as judged by deviation from the total mean of samples (square root of the mean square > 1). Three compounds APM, PPX and VER induced a significant and biologically relevant (induction with more than 50% of control level) induction of reporter expression in both notochord (TukeyHSD, $p=0.0000000$ for all the three chemicals) and muscle (TukeyHSD, $p=0.0000000$, 0.0000555 and 0.0000000, respectively) in the absence of MS-222. In the presence of MS-222, this biologically relevant induction was abolished, albeit statistical significance revealed by TukeyHSD test (*$p<0.05$, ***$p<0.001$). Difference of mean values between a chemical and control (=1) is shown in parenthesis.

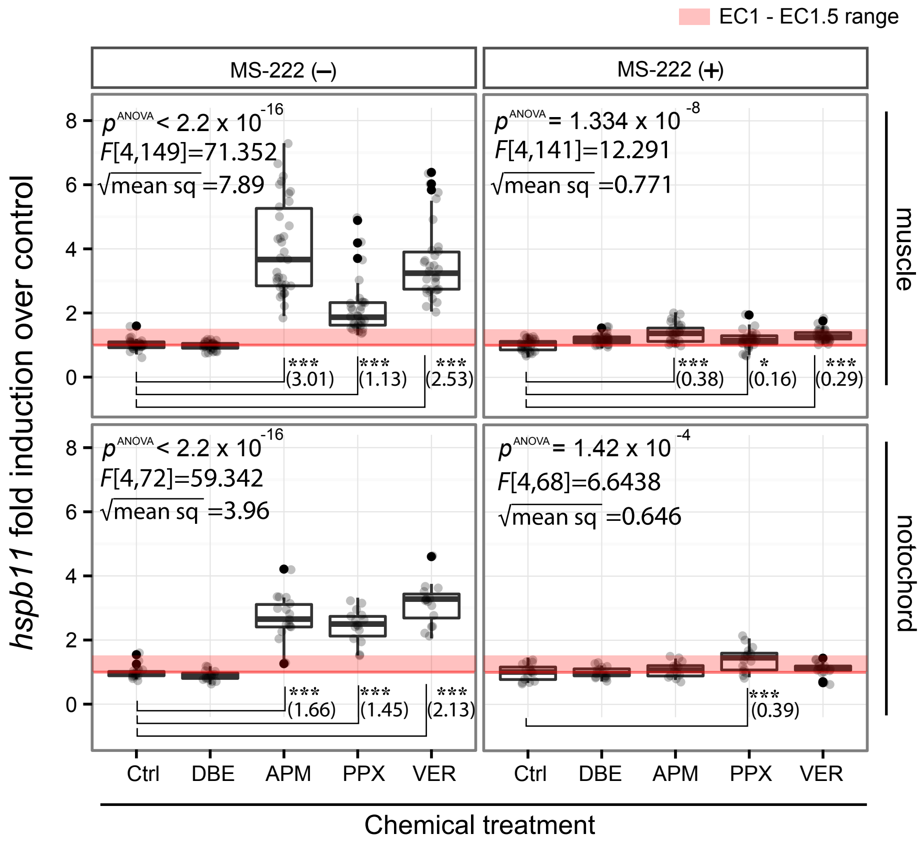

Figure S3

Comparison of chemically-induced *hspb11* transgene response in the muscle and in the notochord

Movie S1

Muscle hyperactivity assessed by confocal time lapse analysis with *Tg([-505/-310]unc45b:GCaMP5A).*

Movie S2

Motility of control embryos at 48 hpf treated with 0.1% DMSO from 9 hpf. Note that embryos do not move in the absence of touch stimuli.

Movie S3

Hyperactive movements observed with 48 hpf embryos treated with 3 µM azinphosmethyl from 9 hpf. Note that embryos show constant shivering and twitching in the absence of touch stimuli.

Table S1.
Mode of action of the compounds used in this study

**Compound**	**Class of Compound**	**Mode of Action**	**Model System**	**Other Affected Organ**	**References**
azinphosmethyl	Organophosphate insecticide	Irreversible AChE inhibitor	Enzymatic assay using Rainbow trout muscle extracts	Muscle, Central, peripheral and autonomic nervous system	a, b, c
propoxur	Carbamate insecticide	Reversible AChE inhibitor	Enzymatic assay using rat brain extracts	Liver, kidney, CNS, muscles	d, e, f
galanthamine	Alkaloid (Treatment for Alzheimer's Disease)	Reversible AChE inhibitor	Enzymatic assay using rat brain and hypophysis extracts	Muscles, CNS, gastrointestinal tract	g, h, i
chlorpyrifos	Organophosphate insecticide	Irreversible AChE inhibitor	Enzymatic assay using zebrafish larval extracts	Muscle, CNS, testies	j, k, l, m
di-*n*-butylphthalate	Phthalates	potential AChE inhibitor, plasticizer	Enzymatic assay using zebrafish larval extracts	CNS, Reproductive system (Endocrine disruptor)	n, o
veratridine	Steroid derived alkaloid	Ion Channel modulator (Persistent opening of the voltage-gated Na+ channel)	Zebrafish	Muscle, CNS, PNS	p, q
flucythrinate	Type II Pyrethroid insecticide	Ion Channel modulator (Persistent opening of the voltage-gated Na+ channel)	Squid	CNS, tongue muscle	r, s, t
methoxychlor	Organochlorine insecticide	Ion Channel modulator (phospholipase C-dependent Ca2+ release)	Renal tubular cells	CNS, Liver, Reproductive system (Endocrine disruptor), muscle	c, u, v, w
esfenvalerate	Type II Pyrethroid insecticide	Ion Channel modulator (Persistent opening of the voltage-gated Na+ channel)	Delta smelt (fish)	CNS, gastrointestinal tract, Reproductive system (Endocrine disruptor)	x, y, z
methylmercury	Organometallic cation	The interactions with sulfhydryl groups, the induction of oxidative stress and the disruption of calcium ion homeostasis	Human embryonic kidney cells	Muscles, Cardiovascular and CNS	aa, ab, ac, ad
4-chlorophenol	Organochloride of phenol	React with glutathione; uncouple oxidative phosphorylation	mouse	skin, liver, immune system	ae
1,2-dibromoethane	Organobromine pesticide	Oxidative phosphorylation disruptor	Rat liver mitochondria	Liver, kidney	ag, ah
2,4-dimethylphenol	Xylenol	Decrease in ATPase activity	Human erythrocytes	Overall growth and development	ai, aj
chlorothalonil	Organochlorine pesticide	Inhibition of NADPH oxidase activity; React with glutathion	Striped bass macrophages	Kidney	b, ak, al

References are followings:

a. Ferrari, A., Venturino, A. & Pechen de D'Angelo, A.M. Muscular and brain cholinesterase sensitivities to azinphos methyl and carbaryl in the juvenile rainbow trout Oncorhynchus mykiss. *Comp Biochem Physiol C Toxicol Pharmacol* **146**, 308-13 (2007).

b. Kamrin, M. (ed.) *Pesticide Profiles: Toxicity, Environmental Impact, and Fate,* (CRC Press, 1997).

c. Kacew M., L.B. (ed.) *Lu's Basic Toxicology: Fundamentals, Target Organs and Rish Assessment* (CRC Press, 2012).

d. Smulders, C.J., Bueters, T.J., Van Kleef, R.G. & Vijverberg, H.P. Selective effects of carbamate pesticides on rat neuronal nicotinic acetylcholine receptors and rat brain acetylcholinesterase. *Toxicol Appl Pharmacol* **193**, 139-46 (2003).

e. Tsitsimpikou, C. et al. Histopathological lesions, oxidative stress and genotoxic effects in liver and kidneys following long term exposure of rabbits to diazinon and propoxur. *Toxicology* **307**, 109-14 (2013).

f. Whitacre, D. (ed.) *Reviews of Environmental Contamination and Toxicology*, (Springer, 2012).

g. Taylor D., P.C., and Kapur S. (ed.) *The Maudsley Prescribing Guidelines in Psychiatry,* (WILEY Blackwell, 2015).

h. Svoboda, Z. et al. Galantamine antiacetylcholinesterase activity in rat brain influenced by L-carnitine. *Biomed Pap Med Fac Univ Palacky Olomouc Czech Repub* **149**, 335-7 (2005).

i. Turiiski, V.I., Krustev, A.D., Sirakov, V.N. & Getova, D.P. In vivo and in vitro study of the influence of the anticholinesterase drug galantamine on motor and evacuative functions of rat gastrointestinal tract. *Eur J Pharmacol* **498**, 233-9 (2004).

j. Yen, J., Donerly, S., Levin, E.D. & Linney, E.A. Differential acetylcholinesterase inhibition of chlorpyrifos, diazinon and parathion in larval zebrafish. *Neurotoxicol Teratol* **33**, 735-41 (2011).

k. Thivakaran, T., Gamage, R., Gunarathne, K.S. & Gooneratne, I.K. Chlorpyrifos-induced delayed myelopathy and pure motor neuropathy: a case report. *Neurologist* **18**, 226-8 (2012).

l. Elsharkawy, E.E., Yahia, D. & El-Nisr, N.A. Chlorpyrifos induced testicular damage in rats: ameliorative effect of glutathione antioxidant. *Environ Toxicol* **29**, 1011-9 (2014).

m. Colombo, A., Orsi, F. & Bonfanti, P. Exposure to the organophosphorus pesticide chlorpyrifos inhibits acetylcholinesterase activity and affects muscular integrity in Xenopus laevis larvae. *Chemosphere* **61**, 1665-71 (2005).

n. Xu, H. et al. Effects of di-n-butyl phthalate and diethyl phthalate on acetylcholinesterase activity and neurotoxicity related gene expression in embryonic zebrafish. *Bull Environ Contam Toxicol* **91**, 635-9 (2013).

o. Cirillo, T. et al. Exposure to Di-2-Ethylhexyl Phthalate, Di-N-Butyl Phthalate and Bisphenol A through Infant Formulas. *Journal of Agricultural and Food Chemistry* **63**, 3303-3310 (2015).

p. Roostalu, U. & Strahle, U. In vivo imaging of molecular interactions at damaged sarcolemma. *Dev Cell* **22**, 515-29 (2012).

q. Wang, G.K. & Wang, S.Y. Veratridine block of rat skeletal muscle Nav1.4 sodium channels in the inner vestibule. *J Physiol* **548**, 667-75 (2003).

r. Brown, L.D. & Narahashi, T. Modulation of nerve membrane sodium channel activation by deltamethrin. *Brain Res* **584**, 71-6 (1992).

s. Davies, T.G., Field, L.M., Usherwood, P.N. & Williamson, M.S. DDT, pyrethrins, pyrethroids and insect sodium channels. *IUBMB Life* **59**, 151-62 (2007).

t. Ahdab, R., Ayache, S.S., Maltonti, F., Brugieres, P. & Lefaucheur, J.P. Motor Neuron Disorder with Tongue Spasms Due to Pyrethroid Insecticide Toxicity. *Neurology* **76**, 196-197 (2011).

u. Chedrese, P.J. & Feyles, F. The diverse mechanism of action of dichlorodiphenyldichloroethylene (DDE) and methoxychlor in ovarian cells in vitro. *Reprod Toxicol* **15**, 693-8 (2001).

v. Cheng, H.H. et al. Effect of Methoxychlor on Ca2+Movement and Viability in MDCK Renal Tubular Cells. *Basic & Clinical Pharmacology & Toxicology* **111**, 224-231 (2012).

w. Grow, W.A. & Eroschenko, V.P. The pesticide methoxychlor disrupts the fusion of myoblasts into myotubes in skeletal muscle cell culture. *Toxicology and Applied Pharmacology* **179**, 105-110 (2002).

x. Connon, R.E. et al. Linking mechanistic and behavioral responses to sublethal esfenvalerate exposure in the endangered delta smelt; Hypomesus transpacificus (Fam. Osmeridae). *BMC Genomics* **10**, 608 (2009).

y. Varró P., S.E., Kovács M., and IVilági I. Nervous System and Gastrointestinal Effects of the Insecticide Esfenvalerate on the Rat: An Ex Vivo Study. *Agricultural Sciences* **5**, 365-375 (2014).

z. Adelsbach TL., a.T.R. Chemistry and fate of fenvalerate and esfenvalerate. *Rev Environ Contam Toxicol.* **176**, 137-54 (2013).

aa. Peng, S., Hajela, R.K. & Atchison, W.D. Effects of methylmercury on human neuronal L-type calcium channels transiently expressed in human embryonic kidney cells (HEK-293). *J Pharmacol Exp Ther* **302**, 424-32 (2002).

ab. Kang, M.S. et al. Methylmercury-induced toxicity is mediated by enhanced intracellular calcium through activation of phosphatidylcholine-specific phospholipase C. *Toxicology and Applied Pharmacology* **216**, 206-215 (2006).

ac. Usuki, F., Yasutake, A., Matsumoto, M., Umehara, F. & Higuchi, I. The effect of methylmercury on skeletal muscle in the rat: a histopathological study. *Toxicol Lett* **94**, 227-32 (1998).

ad. Fernandes Azevedo, B. et al. Toxic effects of mercury on the cardiovascular and central nervous systems. *J Biomed Biotechnol* **2012**, 949048 (2012).

ae. Agency for toxic substances and disease registry, U.S. Department of health and human services. Toxicological profile for chlorophenols. (1999).

ag. Thomas, C., Will, Y., Schoenberg, S.L., Sanderlin, D. & Reed, D.J. Conjugative metabolism of 1,2-dibromoethane in mitochondria: disruption of oxidative phosphorylation and alkylation of mitochondrial DNA. *Biochem Pharmacol* **61**, 595-603 (2001).

ah. U.S. Environmental Protection Agency Washington DC. Toxicological review of 1,2-dibromoethane (CAS No. 106-93-4) in support of summary information on the integrated risk information system (IRIS). (2004).

ai. Duchnowicz, P., Szczepaniak, P. & Koter, M. Erythrocyte membrane protein damage by phenoxyacetic herbicides and their metabolites. *Pesticide Biochemistry and Physiology* **82**, 59-65 (2005).

aj. Holcombe, G.W., Phipps, G.L. & Fiandt, J.T. Effects of phenol, 2,4-dimethylphenol, 2,4-dichlorophenol, and pentachlorophenol on embryo, larval, and early-juvenile fathead minnows (Pimephales promelas). *Arch Environ Contam Toxicol* **11**, 73-8 (1982).

ak. Baier-Anderson, C. & Anderson, R.S. Suppression of superoxide production by chlorothalonil in striped bass (Morone saxatilus) macrophages: the role of cellular sulfhydryls and oxidative stress. *Aquat Toxicol* **50**, 85-96 (2000).

al. Tillman, R.W., Siegel, M.R., Long, J.W. Mechanism of action and fate of the fungicide chlorothalonil (2, 4, 5, 6-tetrachloroisophthalonitrile) in biological systems: I. Reactions with cells and subcellular components of Saccharomyces pastorianus. *Pestic. Biochem. Phys.* **3**, 160-167 (1973).

Table S2

Summary of the effects of toxicants at 48 hpf on *hspb11* reporter expression, motility and birefringence.

Compound	*hspb11* reporter, EC1.5 (μM)	Motility, EC50 (μM)	Lethality, LC50 (μM)	Birefringence[e]
azinphosmethyl[a]	0.1986	1.17	[d]	reduced
propoxur[a]	51.79	14.6	1,959	reduced
galanthamine[a]	1,043	1,088	[d]	reduced
chlorpyrifos[a]	4.374	4.08	84.39	reduced
di-*n*-butylphthalate[c]	4.072[d]	3.70	[d]	reduced
veratridine[b]	94.66	52.13	[d]	reduced
methylmercury[c]	0.5383	0.0327	[d]	reduced
flucythrinate[b]	m.f.	0.781 [d]	[d]	reduced
esfenvalerate[b]	m.f.	0.214	[d]	reduced
methoxychlor[b]	14.30[d]	1.655	[d]	reduced
chlorophenol[c]	437.1	89.40	904.5	reduced
1,2-dibromoethane[c]	n.s.	3,427	9,647	normal
2,4-dimethylphenol[c]	n.s.	321.1	1,268	normal
chlorothalonil[c]	n.s.	>0.5[d]	0.519	normal

Mode of action of each compound is designated with [a] for Acetylcholinesterase inhibitors, [b] for channel modulators and [c] for other toxicants. m.f., modelling failure in regression analysis, n.s., not significant difference from control (one-way ANOVA $p<0.0001$). [d]: out of concentration range. [e]: shown in Fig. 4.
